# A Group of Complexes Based on PAMAM and Quantum Dots Used in Clinical Immunoassays

**DOI:** 10.1186/s11671-020-3291-5

**Published:** 2020-04-03

**Authors:** Yang Liu, Lu Hou, Qiong Guo, Mingjin Zhang, Wei Shi

**Affiliations:** 1 Key Laboratory for Molecular Enzymology & Engineering, the Ministry of Education, Jilin University, Changchun, 130012 Jilin China; 2 College of life sciences, Jilin University, Changchun, 130012 Jilin China

**Keywords:** PAMAM, Quantum dots, Clinical immunoassays, FCM

## Abstract

We report a group of complexes used in clinical immunoassays. The complexes include a PAMAM-conjugated goat anti-rabbit IgG and a QDs-conjugated goat anti-mouse IgG. When rabbit anti-antigen and mouse anti-antigen are added, the corresponding antigen will be detected. The experiment, using the complexes, is simple, convenient, short in time, and short in steps. It is also applicable to different experiment methods, like to be used with FCM (flow cytometry), ICC (immunocytochemistry), and IHC (immunohistochemistry) to detect many kinds of antigens.

## Introduction

Quantum dots (QDs) are widely used as luminaries because of their high fluorescence quantum yields, high photostability, and low photobleaching properties. They are also widely used as organic fluorophores in cellular imaging and biotechnological applications. In particular, cadmium-containing QDs (i.e., CdSe and CdTe) have significant advantages because under irradiation of the same wavelength within the range of 430–660 nm, they have a size-dependent emission electromagnetic spectrum [1, 2]. Therefore, antibody-conjugated QDs are the most promising probes for molecular imaging.

The polyamidoamine dendrimer (PAMAM), as one of the most widely and deeply studied dendritic macromolecules, has the following characteristics: a vast number of surface functional groups, a large number of cavities inside the molecules, a nanoscale size, and high biocompatibility. When conjugated with drugs and antibodies, these dendrimers can improve a drug’s solubility and systemic circulation but do not hamper the drug’s biological activity [3]. Therefore, antibodies modified with PAMAM are often used in clinical immunization.

Clinical immunoassays include many methods, such as WB, ELISA, IHC (immunohistochemistry), ICC (immunocytochemistry), and FCM (flow cytometry). Among them, FCM utilizing specific antibody probes perform a rapid analysis of single cell or other biological particles at the cellular and molecular level. Therefore, FCM is one of the most widely used immunoassays. If cell screening is carried out, the use of the corresponding antibody probe is sufficient; however, if small biological molecule screening is desired, such as that of a small protein, virus, or cytokine, the CBA (cytometric bead array) method should be adopted because FCM cannot directly detect small cytokines. The CBA method utilizes dye-labeled beads constructed with specific capture antibodies on the surface. When CBA beads are mixed with the sample and corresponding dye-labeled antibodies, sandwich complexes will form as in ELISA, and the small antigen can be detected by FCM.

**Scheme 1:**
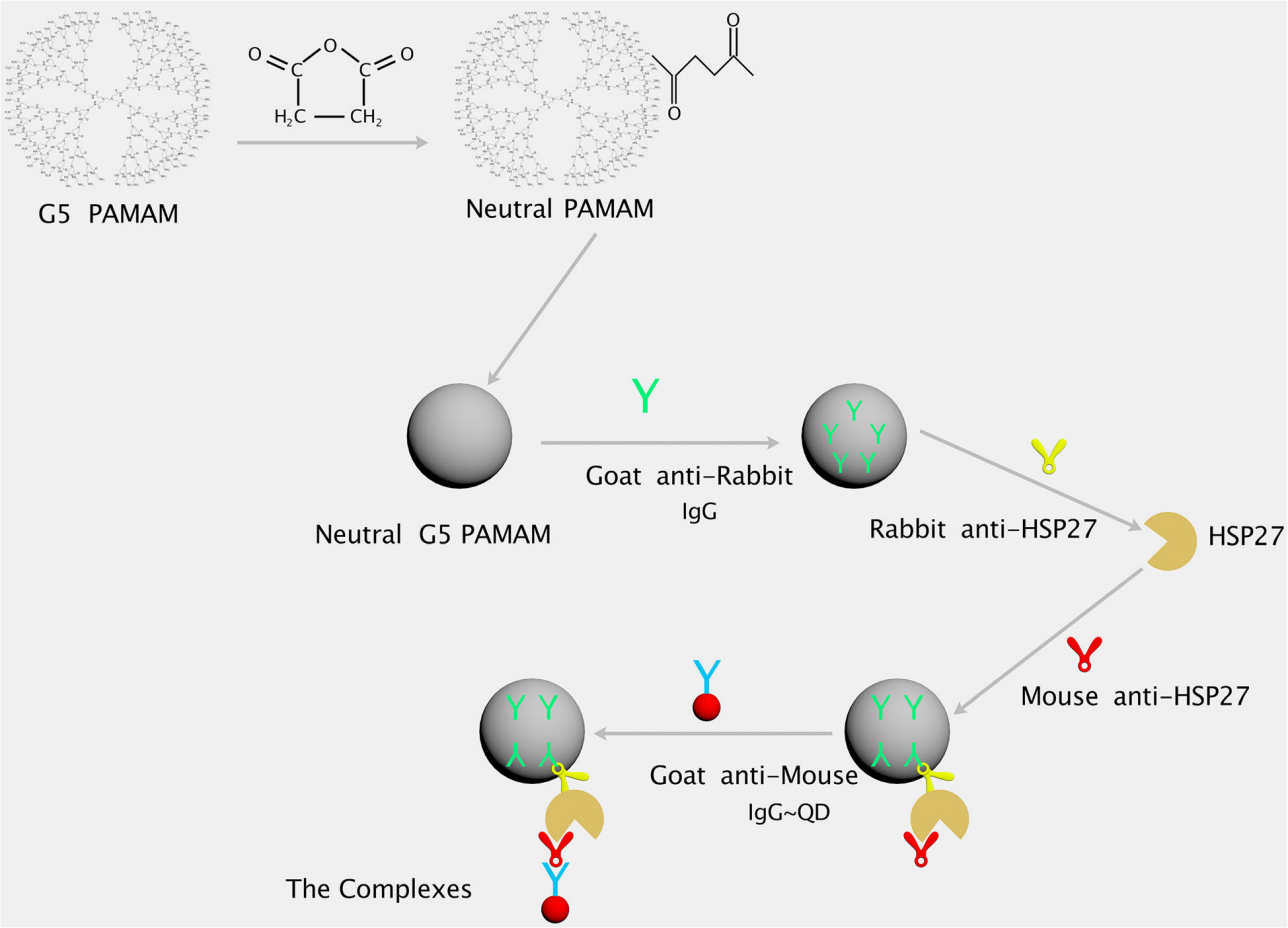
Representation of how the complexes are formed and used by FCM to detect HSP27. First, the G5 amino PAMAM must react with succinic anhydride to form neutral PAMAM

In this study, we present a group of complexes including a PAMAM-conjugated goat anti-rabbit IgG and a QDs-conjugated goat anti-mouse IgG. This group of complexes can be used for FCM, ICC, and IHC. When rabbit anti-antigen and mouse anti-antigen are added, the corresponding antigen will be detected. This model can detect many types of antigens, including small proteins, viruses, and cytokines when the corresponding mouse and rabbit antibodies are present. Scheme 1 shows the complexes used in FCM, we take HSP27 as an example. HSP27 is also known as HSPB1 (heat shock protein family B number 1) and it is an important protein involved in drug resistance, cell growth, apoptosis, tumor occurrence, and metastasis, etc [4, 5]. In this model, the PAMAM-conjugated goat anti-rabbit IgG acts as a carrier and a capture, similar to the CBA (cytometric bead array) beads, allowing the small molecule antigens to be detected by FCM. The QDs-conjugated goat anti-mouse IgG acts as a fluorescent probe. If the antigens are membrane proteins or intracellular proteins, we can use the traditional FCM method to detect the antigens. We only need to add the QDs and not the PAMAM moiety. For intracellular proteins and membrane proteins, cells can be considered as targets that can be directly detected by FCM method; they do not need the PAMAM moiety to constitute a pseudocell. Therefore, we can split the complexes and use them individually.

## Results and Discussion

The group of complexes includes two parts, a PAMAM part and a QDs’ part. The PAMAM part is a PAMAM-conjugated goat anti-rabbit IgG. We used fifth generation amino-terminated PAMAM dendrimers (G5 PAMAM) as a surface stabilizing agent to provide solubility characteristics ideal for aqueous environments, but this dendrimer has a strong positive charge that is harmful for antibody binding. To neutralize the positive charge, we dissolved PAMAM in DMSO and added succinic anhydride (dihydro-2,5-diketotetrahydrofuran) to neutralize the PAMAM amino group. The amino group in PAMAM reacts with succinic anhydride to form amide bonds [6–10]. After the reaction, the product was dialyzed, lyophilized, weighed, and redissolved, the production is nearly neutral and we named the product “N-PAMAM,” which is able to conjugate with goat anti-rabbit IgG. PAMAM, especially G5 PAMAM, contains a large number of cavities, and the IgG may be encapsulated within the void spaces of G5 PAMAM [11]. Goat anti-rabbit IgG was first dissolved in MES buffer (0.1 mol/L, pH 6.0), then EDC and sulfo-NHS (at a molar ratio of 1:1) were added, and the mixture was incubated for 15 min. Finally, N-PAMAM (neutral PAMAM) was added followed by incubation at 4 °C in a shaker bed overnight. This polymer self-assembled in aqueous solution to form polymeric micelles that encapsulated the otherwise insoluble low molecular weight guests [12]; after the reaction, dialysis was required to remove the unreacted materials. Then, the production was lyophilized, weighed, and redissolved in preserving buffer, and finally, the N-PAMAM-conjugated goat anti-rabbit IgG was made.

**Fig. 1:**
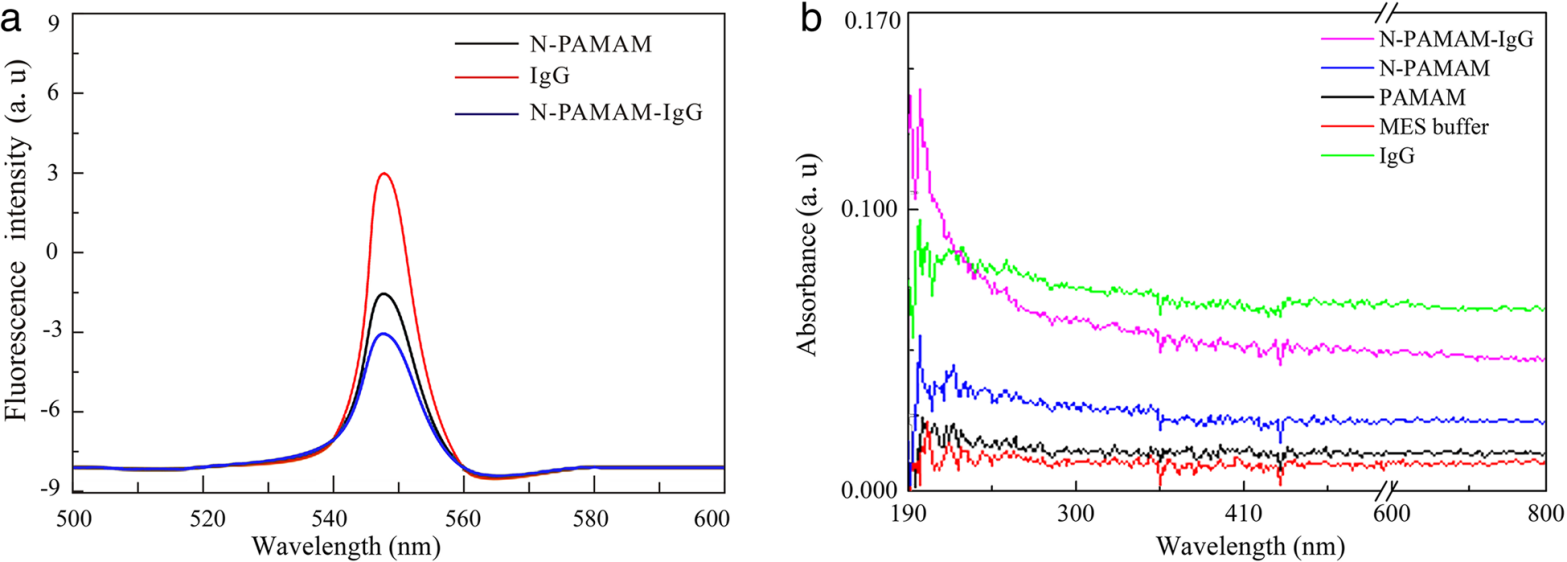
**a** Fluorescence spectra of PAMAM-IgG (goat anti-rabbit) in MES. The excitation wavelength was 548 nm. **b** UV-vis spectra of PAMAM-IgG (goat anti-rabbit) in MES and emission spectra over a range of wavelengths

**Fig. 2:**
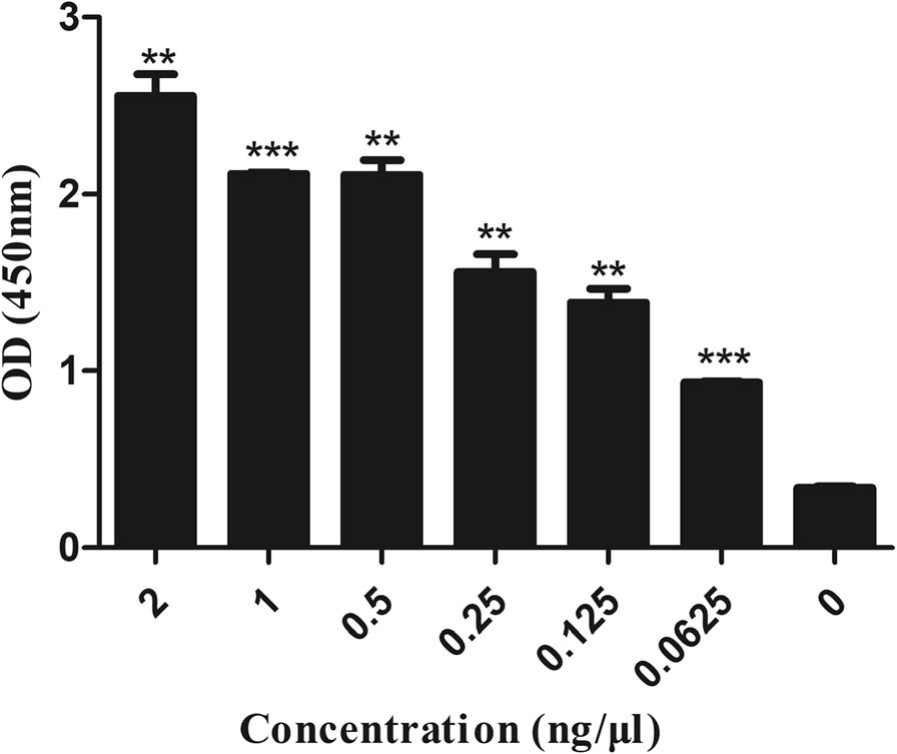
ELISA method to detect N-PAMAM-IgG (goat anti-rabbit) antibody resistance. N-PAMAM-IgG was used as an antigen and diluted to different concentrations. Rabbit IgG-HRP was used as an antibody probe to detect the resistance of N-PAMAM-IgG. The absorbance measurement was taken at a wavelength of 450 nm

The fluorescence and UV-vis spectra of N-PAMAM-IgG (goat anti-rabbit) in MES are shown in Fig. 1. Compared with N-PAMAM (neutral PAMAM) and IgG, the fluorescence emission peak of N-PAMAM-IgG was of the lowest intensity. The UV-vis spectrum shows that compared with IgG, MES buffer, PAMAM, and N-PAMAM, only N-PAMAM-IgG had an absorption peak at a wavelength of 200 nm. Whether the fluorescence spectrum or UV-vis spectrum, N-PAMAM-IgG shows different characteristics compared with N-PAMAM and IgG, which indirectly proves that N-PAMAM and IgG have been combined rather than simply mixed. To detect the antibody activity of N-PAMAM-IgG, ELISA was carried out. As shown in Fig. 2, the IgG (goat anti-rabbit) antibody resistance was not lost upon coupling with N-PAMAM.

The QDs’ part is a QDs-conjugated goat anti-mouse IgG. Antibody-conjugated QDs are usually formed by cross-coupling reactions, where antibody molecules bind to functional groups such as carboxyl groups or amino groups on the QDs’ surface [13, 14]. In this study, we used core/shell CdSe/ZnS water-soluble quantum dots stabilized with carboxyl ligands and the carbodiimide coupling reaction using EDC (1-ethyl-3-(3- dimethylaminopropyl)-carbodiimide hydrochloride). This method is one of the most popular methods for conjugating an antibody to the surface of QDs [15–17]. The CdSe/ZnS QDs’ micelles were first incubated in the presence of EDC and sulfo-NHS in borate buffer (5 mM BS, pH 7.2). Then, goat anti-mouse IgG was added, and the mixture was incubated at 4 °C in a shaker bed overnight in the dark. Finally, 10% BSA (bovine serum albumin) was added to block the unreacted QDs, and the production was purified by successive centrifugation and redissolving to remove the unreacted materials. The antibody activity of the QDs-conjugated IgG (goat anti-mouse) can keep for 3 months after storage at 4 °C in the dark.

**Fig. 3:**
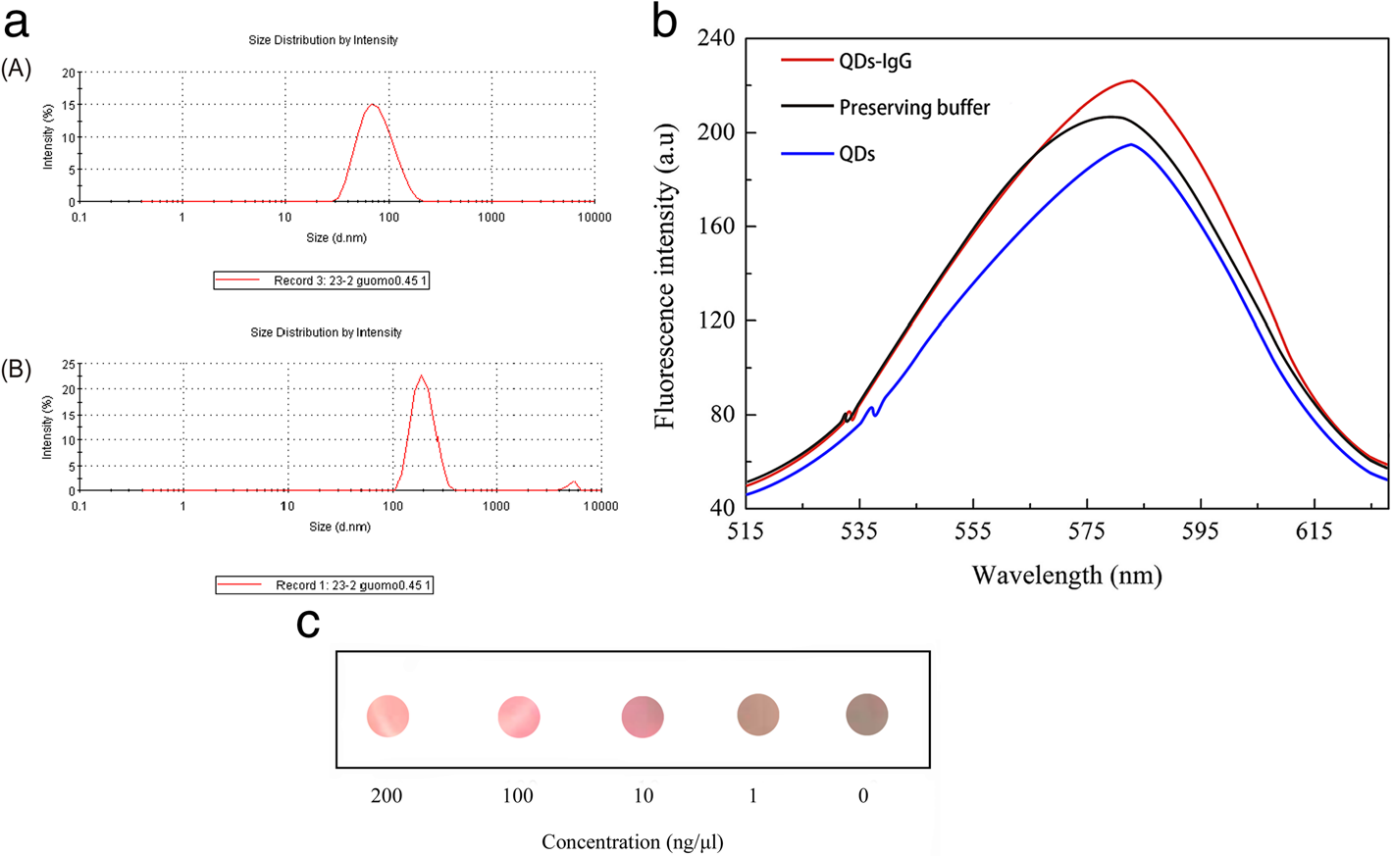
**a** Distribution of hydrodynamic diameter of the QDs and QDs-conjugated IgG (goat anti-mouse). (**a**) QDs’ diameters and (**b**) QDs-conjugated IgG (goat anti-mouse). The average diameter of the QDs is 64.87 nm, and that of QDs-IgG is 211.4 nm, detected by DLS. **b** Fluorescence spectra of QDs-IgG (goat anti-mouse), QDs and preserving buffer (2.5 mM BS, pH 8.0, 0.1% BSA). The QDs-IgG fluorescence emission peak was the same as that of the QDs, indicating that the coupling of IgG to QDs did not alter the QDs’ optical characteristics. **c** ELISA method to detect the antibody resistance of QDs-IgG (goat anti-mouse), the PVDF membrane was assessed with UV

The particle size of the QDs-IgG (goat anti-mouse) and QDs in preserving buffer (2.5 mM BS, pH 8.0, 0.1% BSA) are shown in Fig. 3 a. After the coupling reaction, the diameter of the products obviously increased, and the products had good homogeneity and stability. These characteristics are the key to their use as a detection probe. The fluorescence spectra of the QDs-IgG and QDs are shown in Fig. 3b, and as shown in Fig. 3a, the production diameter increased shows that the IgG was combined with the QDs, but the QDs-IgG fluorescence emission peak was the same as the QDs. This result means that the coupling of IgG and QDs did not alter the optical characteristics of the QDs, which is useful for their application in immunoassays. To detect the antibody activity of the QDs-IgG, we used an ELISA method with a PVDF membrane. The mouse anti-AKT antibody was diluted to concentrations of 1 μg/ml, 10 μg/ml, 100 μg/ml, and 200 μg/ml, and the four concentration gradient antigens were hybridized with QDs-IgG (goat anti-mouse) at the same concentration of 0.1 mg/ml. After hybridization for 40 min at 37 °C in the dark, the PVDF membrane was washed with PBST (pH 6.0, 0.1% Tween 20) 3 times, and the PVDF membrane was irradiated with UV light. The fluorescence intensity was shown in Fig. 3c, the fluorescence intensity increased with increasing antigen concentration, indicating that QDs-IgG has high antibody activity.

**Fig. 4:**
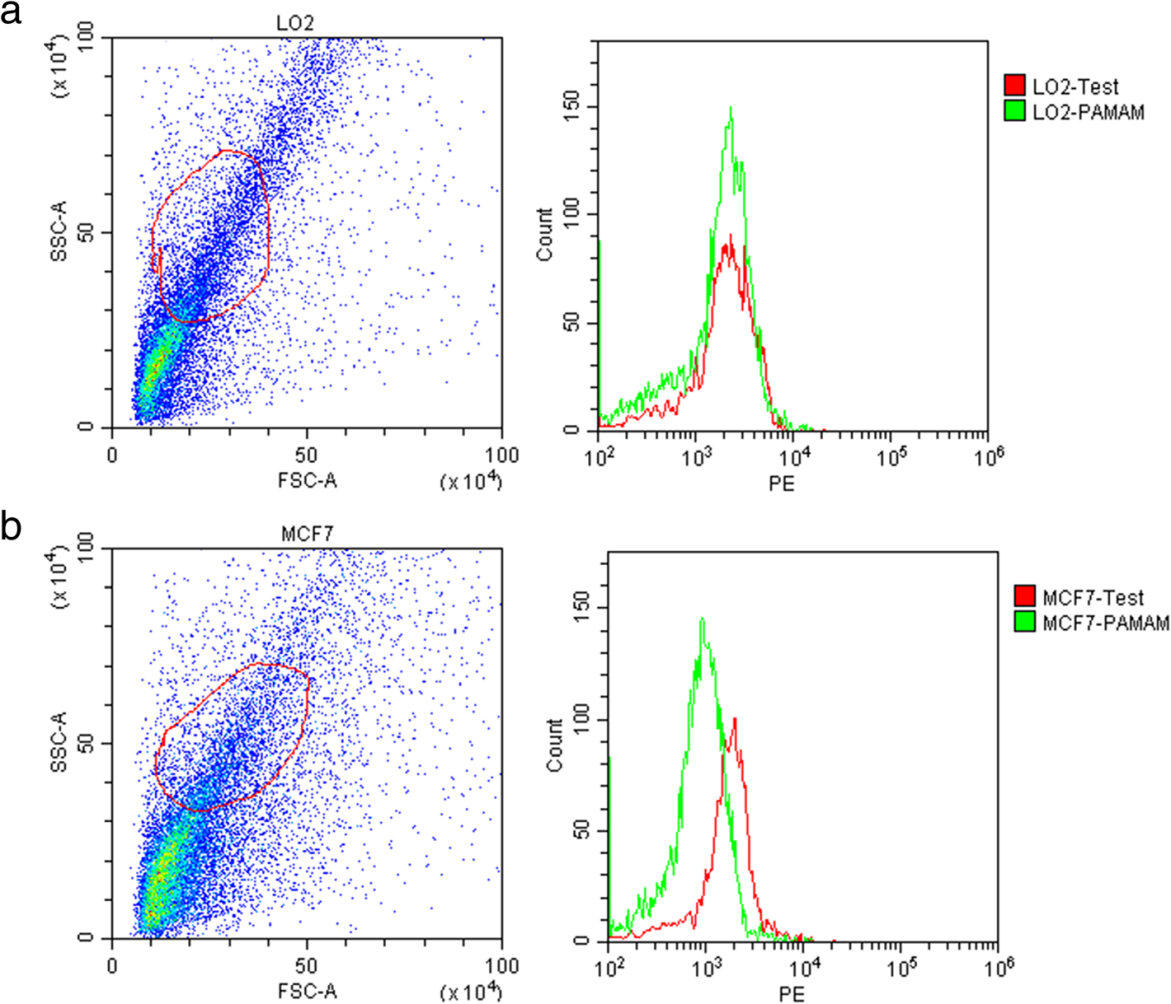
Fluorescence analysis of HSP27 by flow cytometry. **a** L02 cells. **b** MCF-7 cells. The test group was incubated with N-PAMAM-IgG and QDs-IgG, PAMAM group as the control, which was only incubated with N-PAMAM-IgG to form a pseudocell for micromolecule protein detected by FCM. When the two curves almost overlap, it can be determined that there is no HSP27 in the sample; therefore, whether QDs-IgG is added or not, the fluorescence intensity will not change

This section introduces how to utilize this group of complexes in FCM. As mentioned above, we can detect many types of antigens, such as small proteins, viruses, and cytokines [18], and we used the HSP27 protein as an example, now let us go over the process in detail. HSP27 is located in the cytoplasm and is secreted in large quantities in tumor cells, especially in lung cancer, pancreatic cancer, urinary epithelial cancer, kidney cancer, breast cancer, and melanoma skin cancer. Since small part of HSP27 is extracellular secreted, we need to lyse the cell to extract the protein. In this experiment, we selected two cell lines, MCF-7 (human breast cancer cells) as the test group and L02 (normal human liver cells) as the control group. The cells were lysed to extract the intracellular proteins. The total proteins were collected then rabbit anti-HSP27 and mouse anti-HSP27 were added according to the protein concentration, followed by incubation at 37 °C for 50 min. Then N-PAMAM-IgG was added to the mixture, the mixture was incubated at 37 °C for 30 min, and then divided into two equal groups: a test group and a PAMAM group. Finally, QDs-IgG was added to the test group and incubated at 37 °C for 30 min in the dark. Figure 4 shows the fluorescence analysis for the two groups of cells by flow cytometry. The fluorescence intensity of the test curve was much higher than that of the PAMAM curve in the MCF-7 group, while in the L02 group, the two curves almost overlapped. When HSP27 exists in cell extracts, N-PAMAM-IgG and QDs-IgG will indirectly bind together to form a sandwich combination in the presence of rabbit anti-HSP27 and mouse anti-HSP27. If HSP27 does not exist, only a small amount of QDs-IgG will bind to N-PAMAM-IgG by nonspecific adsorption. Therefore, if the fluorescence intensity of the test curve is higher than that of the control PAMAM curve, HSP27 is contained in the sample. Figure 4 shows that MCF-7 cells secreted more HSP27 protein than L02 cells.

**Fig. 5:**
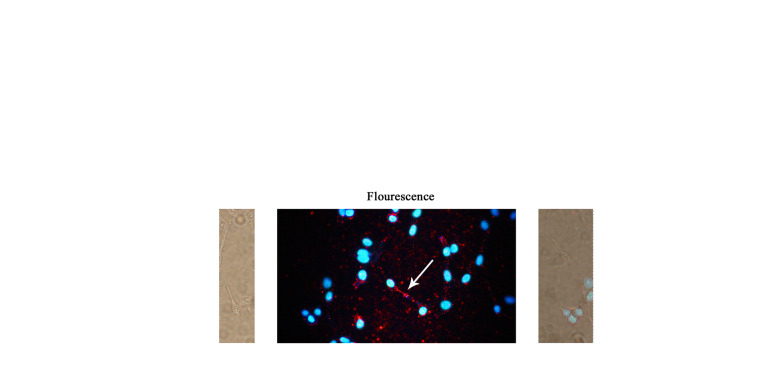
**a** Fluorescence analysis for β-actin in HeLa cells. The β-actin group was incubated with mouse anti-β-actin and QDs-IgG (goat anti-mouse), and the control group was incubated with BSA and QDs-IgG. The control group can allow for the exclusion of the background fluorescence caused by the nonspecific adsorption of QDs-IgG. **b** Fluorescence microscope images of HeLa cells under UV-vis irradiation. DAPI stained the cell nuclei and emitted blue fluorescence; QDs-IgG indirectly combined with β-actin and emitted red fluorescence. Scale bar 20 μm

As mentioned above, these complexes can detect secreted intracellular proteins by FCM; in principle, these complexes can be applied to any kind of antigen. Furthermore, the two parts of the combination can be used separately. If the antigen we want to detect is located in the cell or on the cell surface, we can only use the QDs’ part. For example, β-actin is one of the main components of the cytoskeleton located in cells and widely exists in eukaryotic cells. Figure 5a shows the fluorescence analysis only used QD part for β-actin in HeLa cells by FCM. HeLa cells were washed and treated with methanol to enhance the penetration of the cell membrane and then divided into two equal groups. The β-actin group was incubated with mouse anti-β-actin at 37 °C for 30 min, and the control group was incubated with BSA. After washing with PBS, both groups were incubated with QDs-IgG (goat anti-mouse) at 37 °C for 30 min. After washing with PBS twice, both groups were detected by FCM. The fluorescence intensity of the β-actin curve was much higher than that of the control curve, which indicated that the HeLa cells contained the β-actin protein.

In addition, these complexes can also be applied to ICC. However, for ICC, it is required that the target antigen be located in the cell or on the cell surface and be present in large quantities [19]; otherwise, the experimental target will be difficult to distinguish from the background. We took the β-actin protein for example and seeded HeLa cells in a 10 cm cell plate and placed a microscope coverslip in it, use methanol to immobilize cells. The cells were then incubated with mouse anti-β-actin (PBS, 1% BSA) at 37 °C for 1 h, washed twice with PBST, and incubated with QDs-IgG (goat anti-mouse) at 37 °C in the dark for 1 h. After washing twice with PBST and staining the cell nucleus with DAPI, the fluorescence was detected with a fluorescence microscope [20, 21]. Figure 5b shows the fluorescence microscope images of the HeLa cells. The advantage of QDs as a fluorescent dye is that the QDs’ signal and the DAPI signal can be simultaneously observed under the irradiation of the ultraviolet channel; therefore, according to the nucleus, the target can be obviously located. In this experiment, β-actin is cytoskeleton proteins around the cell nucleus and can be obviously observed.

## Conclusions

In conclusion, we demonstrated the application of the complexes used in clinical immunoassays, such as FCM and ICC. The group of complexes includes a PAMAM-conjugated goat anti-rabbit IgG and a QDs-conjugated goat anti-mouse IgG. When rabbit anti-antigen and mouse anti-antigen were added, the corresponding antigen was detected. These complexes are advantageous due to their broad spectrum, high biocompatibility, high light stability, and low biological toxicity. This complex is a universal model that only requires the corresponding primary antibodies to be changed. In this article, we used the complexes to detect HSP27 and β-actin, but in theory, this process can be applied to any type of antigen. Futhermore, we can choose the detection method according to the characteristics of the antigens, and we can also split the complexes and use them individually according to the actual needs. This method also allows small molecules, such as small proteins and viruses, to be detected by flow cytometry. We believe that with appropriate improvement, it also can be applied to other methods, like immunoflurescence (IF), western blot (WB), and lateral-flow immunochromatographic strip (LCS).

## Methods/Experimental

### Materials

CdSe/ZnS quantum dots (water soluble QDs with carboxyl groups, cas no. N/A) were purchased from NEPQD, China. PAMAM (fifth generation amino terminated, Lot no. CYD-150A) was purchased from CYD, China. Goat anti-rabbit IgG (ab6702) and mouse anti-β-actin (ab8226) were purchased from Abcam, UK; mouse anti-HSP27 (BF0624) and rabbit anti-HSP27 (AF6082) were purchased from Affinity, USA; goat anti-mouse IgG (bs0296G) was purchased from Bioss, China; EDC and sulfo-NHS were purchased from Thermo Fisher; DMEM and fetal bovine serum were purchased from Gibco; and PVDF membranes (0.2 μm) were purchased from Millipore. All other chemical reagents were of analytical reagent grade.

### Preparation of N-PAMAM-IgG (goat anti-rabbit)

- Preparation of N-PAMAM (neutral PAMAM): PAMAM (G5 amino-terminated, average MW 28826) (60 mg, 2.081 mM) was dissolved in DMSO (2 ml) and succinic anhydride (dihydro-2,5-diketotetrahydrofuran) (0.266 g, 2.66 mM) was added to neutralize the amino groups. Because one mole of G5 PAMAM macromolecules has 128 moles of amino groups, 60 mg of PAMAM contains 0.266 mM amino groups, and the molar ratio for PAMAM amino groups and succinic anhydride is approximately 1:10. Then, the mixture was blended in a shaker bed at 100 rpm for 4 h, after which the solution was dialyzed through a 3500 MWCO dialysis membrane for 24 h, and N-PAMAM was obtained after lyophilization.
- Preparation of N-PAMAM-IgG (goat anti-rabbit): formulated MES buffer (100 mM, pH 6.0), then take 100 μl MES buffer into a tube, add 20 μM EDC, and 20 μM sulfo-NHS then agitate using a vortex at a low speed. Add 35.7 μl IgG (pH, 0.5 μM) and agitate by vortex, after that add 19 μg N-PAMAM and incubate at 4 °C for overnight, finally the solution will be dialyzed through a 3500MWCO and a 8000MWCO dialysis membrane for 3 days, after lyophilizate the N-PAMAM-IgG will be gain.

### Preparation of QDs-IgG (goat anti-mouse)

- Formulation of BS buffer:

1. Prepare borax buffer (50 mM): weigh 19.07 g of borax and dissolve in 1 L of ultrapure water.
2. Prepare boric acid buffer (50 mM): weigh 3.09 g of boric acid and dissolve in 1 L of ultrapure water.
3. pH 8.0 BS buffer and pH 7.2 BS buffer were prepared by mixing the above two solutions.
4. Washing buffer, also used as preserving buffer, was prepared by diluting the pH 8.0 BS buffer to a concentration of 5 mM and adding 0.1% Tween 20.
5. Activation solution was prepared by diluting the pH 7.2 BS buffer to a concentration of 5 mM and adding 0.1% Tween 20.
6. EDC buffer was made by dissolving 0.27 g of EDC in 5 ml of activation solution, and sulfo-NHS buffer was made by dissolving 0.378 g of sulfo-NHS in 5 ml of activation solution.

b) Preparation of QDs-IgG (goat anti-mouse):

First, 450 μl of QDs (CdSe/ZnS, 5 mg/ml) was dissolved in 2.25 ml activation solution, then 150 μl of EDC buffer and 150 μl of sulfo-NHS buffer were added, and the solution was sonicated on ice for 5 min. Second, the solution was centrifuged at 12,000 rpm for 5 min to remove the supernatant, and the precipitate was dissolved in 1.2 ml of washing buffer. After 30 min of ultrasonic mixing, 100 μg of IgG antibody was added, then the solution was incubated overnight in a shaker bed at a temperature of 4 °C. Third, 150 μl of 10% BSA was added followed by incubation at 30 °C for 30 min and centrifugation at 12,000 rpm for 2 min to remove supernatant. The precipitate was redissolved in 1 ml of washing buffer then centrifugeted at 12,000 rpm for 2 min, this step was repeated and finally resolved the precipitate in 1 ml of preserving buffer. At the end, 10% BSA was added; the mixture was mixed with full ultrasonic shock mixing and centrifuged at 820 g/min to remove the precipitate, with the supernatant containing QDs-IgG. QDs-IgG should be stored at 4 °C in the dark for 3 months. It should be noted that the samples can only be stored at 4 °C, and cannot be frozen even with glycerin; otherwise, it will be caused a large number of quantum dots gathered into clusters, forming precipitation that could not be washed away with PBST, this lead to serious background fluorescence.

### FCM Analysis for HSP27 Protein

HSP27 is located in the cytoplasm and is secreted in large quantities in tumor cells. In this experiment, we selected two cell lines, MCF-7 (human breast cancer cells) as the test group and L02 (normal human liver cells) as the control group. The two groups of cells were seeded in a 10 cm culture dish and incubated in Dulbecco’s modified Eagle’s medium (DMEM) with 10% fetal bovine serum (FBS) at 37 °C. When cells covered 80% of the plate, the cells were digested with trypsin and washed twice with PBS. Then, the cells were resuspended in 1 ml of PBS. Counted the cells with a hemocytometer, T-PER cell lysis buffer was added according to the cell concentration, after that the intracellular protein was extracted from the cell lysate. Then, the cell lysate was centrifuged at 14000 rpm for 10 min to collect the supernatant, and the protein concentration was measured by the BCA method, according to the protein concentration, the rabbit anti-HSP27 and mouse anti-HSP27 were added and incubated at 37 °C for 50 min. In this experiment, the protein concentration of L02 was approximately 0.2 mg/ml and MCF-7 was 0.25 mg/ml, and the volume of both was 500 μl. The two antibodies, rabbit anti-HSP27 and mouse anti-HSP27, were added to the cells (0.625 μl to MCF-7 and 0.5 μl to L02 cells).

Then, 1 mg/ml N-PAMAM-IgG was added to the mixtures followed by incubation at 37 °C for 30 min; 6.25 μl was added to the MCF-7 group, and 5 μl was added to the L02 group. After incubation, the production was divided into two equal groups, the test group and the PAMAM group. Finally, QDs-IgG was added to the test group, which was incubated at 37 °C for 30 min in the dark. Then, the two groups of cells were analyzed by flow cytometry.

### FCM Analysis for the β-actin Protein

β-actin is an intracellular protein that is one of the main components of the cytoskeleton and widely exists in eukaryotic cells. HeLa cells were seeded in a 10 cm culture dish and incubated in DMEM with 10% FBS at 37 °C for 2 days, then the supernatant was removed and the cells were washed twice with PBS. The cells were digested with trypsin, after which the cells were centrifuged at 800 rpm for 3 min to remove the supernatant, and 1 ml of cold methanol was added to resuspend the cells. After 5 min, the cells were centrifuged at 800 rpm for 3 min to remove the supernatant, and the cells were resuspended in 1 ml of PBS. This step was repeated, and the HeLa cells were divided into two equal groups. The test group was incubated with mouse anti-β-actin at 37 °C for 30 min, and the control group was incubated with BSA (bovine serum albumin). In this experiment, mouse anti-β-actin was dissolved in PBST (1% BSA, 5 μg/ml), and 100 μl of IgG was added to 300 μl of the test group, and an equivalent amount of BSA was added to the control group. After half an hour of incubation, both groups were incubated with QDs-IgG (goat anti-mouse) at 37 °C for 30 min. After washing twice with PBS, both groups were assessed by FCM.

### ICC Analysis for the β-actin Protein

HeLa cells were seeded in a 10 cm culture dish and incubated in DMEM with 10% FBS at 37 °C for 1 day. Then, several sterilized coverslips were placed into the cell dish, and the cells were further cultured for 2 days. The coverslips were removed and washed twice with PBS, then the coverslips were incubated in 400 μl of methanol for 20 min at room temperature. The coverslips were washed 3 times with PBS. Then, blocking buffer was prepared with PBST (0.1% Tween 20), 22.52 mg/ml glycine, and 10% BSA. A coverslip was placed into blocking buffer and incubated for 30 min at room temperature. Then, the blocking buffer was removed, and a coverslip was incubated with mouse anti-β-actin (PBS, 1% BSA) at 37 °C for 1 h, washed twice with PBST, and incubated with QDs-IgG (goat anti-mouse) at 37 °C for 1 h in the dark. After washing twice with PBST and staining the cell nucleus with DAPI for 2 min, the coverslip was rinsed once with PBS and once with water for visualization under a fluorescence microscope.

## Data Availability

All data are fully available without restriction.

## Abbreviations

CBA: Cytometric bead array
DMEM: Dulbecco’s modified Eagle’s medium
FBS: Fetal bovine serum
FCM: Flow cytometry
ICC: Immunocytochemistry
N-PAMAM: Neutral PAMAM
PAMAM: The polyamidoamine dendrimer
QDs: Quantum dots
